# Sense of Relational Entitlement and Couple Outcomes: The Mediating Role of Couple Negotiation Tactics

**DOI:** 10.3390/bs13060467

**Published:** 2023-06-03

**Authors:** Octav-Sorin Candel

**Affiliations:** Faculty of Psychology and Educational Sciences, Alexandru Ioan Cuza University of Iaşi, 700554 Iaşi, Romania; octav.candel@uaic.ro

**Keywords:** sense of relational entitlement, negotiation, couple satisfaction, couple conflict

## Abstract

Previous research shows a link between the sense of relational entitlement and various couple outcomes. However, the mechanisms linking these variables are less discussed. With this study, the aim was to test the associations between individuals’ excessive and restricted sense of relational entitlement and their levels of couple satisfaction and conflict. In addition, it was tested whether the use of different negotiation tactics (cooperative and competitive) mediated the links. Six hundred and eighty-seven adults (55.2% women) participated in this study. Mediation analyses showed that a restricted sense of relational entitlement is associated with couple satisfaction and conflict through higher competitive negotiation use. Additionally, an excessive sense of relational entitlement is linked with couple satisfaction and conflict through lower cooperative negotiation use. This study has important implications for couples therapy addressing satisfaction issues, showing why and when educating couple interactions, especially those regarding negotiation, can improve relational functioning. Additionally, one’s relational well-being is strongly related to one’s mental health, and the applicability of the findings can be extended to all outcomes of the therapeutic process.

## 1. Introduction

Having fulfilling intimate relationships is beneficial for an individual’s well-being, with couple satisfaction being related to lower depression and higher life satisfaction [1,2,3]. On the contrary, couple conflict correlates with higher levels of depression and negative mood [4]. Thus, studying the factors leading to changes in a couple’s relational quality is crucial when discussing how to keep individuals more satisfied with their relationships and, therefore, safer from mental health issues. Among others, the sense of relational entitlement (SRE) has been proposed as a relevant personal characteristic related to relational quality [5,6]. However, the mechanisms linking it to couple satisfaction and couple conflict have not been sufficiently explored. 

In this current research, I propose the use of different negotiation tactics as mediators between the sense of relational entitlement and couple satisfaction and conflict. Previous studies showed that negotiation is important for couples, both in daily interactions as well as in therapy [7,8,9]. In addition, entitlement was previously linked with the use of more unethical negotiation behaviors [10]. 

### 1.1. The Sense of Relational Entitlement and Couple Relationships

From a theoretical standpoint, Karney and Bradbury, in 1995, proposed the vulnerability-stress-adaption (VSA) model of couple satisfaction [11]. The authors point out that the quality of a couple’s relationship can be influenced by both the stressful events encountered by the partner as well as by their existing vulnerabilities. The SRE can act as one such vulnerability.

Psychological entitlement represents the belief that one deserves more than others as well as the expectation of special treatment. While initially conceptualized as a broad dimension of narcissism, later theories proposed that individuals express various types of entitlement in various life domains [6,12,13]. The concept of SRE was developed in the context of romantic relationships, referring only to those expressions of entitlement appearing in the domain of romantic relationships. SRE can be defined as the extent to which a person expects that their needs and wishes will be fulfilled by their romantic partner, and as a person’s affective and cognitive responses to a romantic partner’s failure to fulfill these needs and hopes [6]. Moreover, recent studies differentiate between adaptive forms and maladaptive forms of entitlement. Among the latter, Tolmacz, in 2011, proposed excessive and restricted entitlement as forms of SRE [14]. In the context of romantic relationships, individuals with excessive entitlement have high expectations from their partners, as they demand the fulfillment of their needs and wishes. However, they are very sensitive when these expectations are violated and can experience regret regarding their relationship. On the contrary, individuals with a restricted SRE suffer from an inhibited expression of their needs and have doubts about whether is fair for them to express their wishes [13].

Tolmacz described the sense of relational entitlement as developing in the context of attachment relationships with childhood caregivers [14]. However, just like the internal working models of attachment adapt over time to different attachment figures, the sense of relational entitlement changes too. Thus, romantic relationships become the central context to study relational entitlement, since the romantic partner is the main attachment figure and source of emotional need fulfillment during adulthood. Previous studies support this perspective and show that the sense of relational entitlement is significantly linked to couple satisfaction [6,15,16,17]. When differentiating between the excessive and restricted types of entitlement, most studies show that both forms of relational entitlement show negative relationships with satisfaction, but these are stronger for the former compared with the latter [18,19]. In addition, other studies show the opposite link between entitlement and couple conflict. People with higher levels of entitlement (both general and relational) also report higher levels of conflict [5,20,21]. 

### 1.2. The Mediating Role of Negotiation Tactics

One key theory used in understanding the use of negotiation in couple relationships is the dual concern theory [22]. Although it originated from the organizational domain, it was later expanded to the domain of couple relationships [23]. The theory proposes that when negotiating, individuals are faced with two concerns, to defend their own interests and to foster cooperative agreement. When they intend to defend their own interest, they use more competitive negotiation tactics. On the contrary, when their shared interest is more important, they use more cooperative tactics. 

Previous studies showed that using cooperative negotiation tactics is associated with higher levels of quality in one’s romantic relationships, while using competitive tactics is associated with lower levels of quality [23]. Similar results were found when testing the association between effective negotiation and relational quality or equity-restoring negotiation strategies and relational satisfaction [24,25]. Moreover, competitiveness can escalate into exceedingly hostile exchanges; thus, the perpetuation of couple conflict is possible when more competitive or equity-resisting tactics are used [25,26,27].

While the outcomes of negotiation have been previously explored, the psychological antecedents of negotiation have received far less attention, especially in the domain of couple relationships. Past studies found that interindividual differences, such as personality traits and attachment orientation, impact negotiation behaviors [28,29]. One study that focused on the relationship between psychological entitlement and negotiation found that highly entitled individuals are more likely to use a forceful negotiation style and endorse more unethical behaviors [10]. Thus, excessively entitled individuals can fall victim to a “social trap”, where one can find forcing or competitive negotiation tactics as alluring because, if successful, they allow the fulfillment of one’s own needs over the partner’s needs. At the same time, such tactics can have a deleterious effect on one’s relationships [30]. Past literature presents scarce information about the negotiation behaviors of restrictedly entitled individuals. However, restricted entitlement is strongly associated with avoidant attachment [14]. By proxy and based on past studies showing that attachment avoidance has strong links with demanding negotiation strategies [31], one can also expect that the relationship’s restricted entitlement and negotiation will show a similar pattern. 

Most previous studies linked general entitlement and negotiation. However, since the level of entitlement can vary between life domains, each of these deserve attention [32]. A gap in the current couple- and family-related literature is that few studies tried to link interindividual differences, such as those in the level of entitlement, to the use of different negotiation tactics and their couple-relevant outcomes. For example, Williams and collaborators (2018) found that the sense of relational entitlement is associated with conflict resolution tactics. However, while there are similarities between conflict resolution tactics and negotiation tactics, the two processes are not the same. Moreover, the authors did not test the levels of couple conflict experienced by the participants. 

### 1.3. The Present Study

This study takes into account the VSA model of couple satisfaction. Based on the aforementioned empirical findings, I tested whether higher levels of excessive SRE are significantly associated with lower couple satisfaction and higher couple conflict (H1). Additionally, I expected that higher levels of restricted SRE are significantly associated with lower couple satisfaction and higher couple conflict (H2). With this study, I explore one possible mechanism linking the sense of relational entitlement (both excessive and restricted), couple satisfaction and conflict. For this, I propose the use of negotiation tactics as a mediating variable. Thus, the use of competitive and cooperative negotiation tactics will mediate the link between an excessive SRE and couple satisfaction and conflict (H3). Finally, the use of competitive and cooperative negotiation tactics will mediate the relationship between a restricted SRE and couple satisfaction and conflict (H4).

## 2. Method

### 2.1. Participants

Six hundred and eighty-seven adults involved in a romantic relationship at the time of the data collection took part in this study. Among them 379 (55.2%) were women and 308 (44.8%) were men. The mean age was 22.97 years old (SD = 3.54, Min = 18, Max = 35) and the mean length of the relationship was 36.31 months (SD = 39.49, Min = 3, Max = 240). In total, 631 participants (91.8%) were not married. Additionally, 403 participants (58.7%) were cohabitating with their partner. Most participants had no children (N = 651, 94.8%), 18 participants had one child (2.6%), 15 participants had 2 children (2.2%) and three participants had 3 children (0.4%). In terms of education status, most participants had a high school degree (N = 478, 69.6%), four of them finished secondary education (0.6%), 178 had a Bachelor’s degree (25.9%), 26 had a Master’s degree (3.8%) and one had a Ph.D. (0.1%). 

### 2.2. Procedure

The research was approved by the Research Ethics Committee at Alexandru Ioan Cuza University. The questionnaires were distributed to students enrolled in two undergraduate programs who also had to invite one other individual to take part in the study. The participants received an online form containing all the questionnaires used to measure the study’s variables and the demographic questions. Participation was voluntary and the students received academic credits for their involvement.

### 2.3. Instruments

The Sense of Relational Entitlement Scale (SRE) [6]. I used the Romanian translation of the original SRE scale to assess the participants’ sense of relational entitlement [15]. The instrument has 18 items and uses a Likert-type scale with answers from 1 (not at all) to 7 (very much). The questionnaire measures three types of SRE, assertive, excessive and restricted. However, previous research showed insufficient theoretical and methodological clarity for the assertive SRE domain [13]. Thus, in the present study, only the excessive (seven items, e.g., “I often feel I deserve to get more than I do in my relationship”) and the restricted (three items, e.g., “I’m often preoccupied with the question of whether I deserve my partner”) dimensions were retained. The scores for these dimensions were computed by averaging the items for each scale. Both subscales demonstrated good levels of internal consistency. For excessive relational entitlement, α = 0.88; for restricted relational entitlement, α = 0.79.

Negotiation tactics. To measure negotiation tactics, I used a scale created by Livingstone [23], who adapted De Dreu and Boles’s scale [33]. This instrument contains a list of negotiation heuristics referring to retrospective accounts of negotiation tactics used by individuals in their romantic relationships. The participants had to follow the guideline “Please describe the extent to which you utilized any of the following types of tactics while you are negotiating the roles and involvement in various joint activities (e.g., household activities, free time, time spent with friends or family, etc.) with your spouse/partner” and answer to each question on a scale from 1 (never) to 5 (always). The instrument has two sub-scales, each containing eight items: competitive negotiation tactics (“Did you feel that your partner’s loss was your gain”, “Winner takes all”) and cooperative negotiation tactics (“Were willing to compromise”, “We played fair”). For both sub-scales, the total was computed by averaging the items. The scales showed adequate internal consistency. For cooperative negotiation, α = 0.65; for competitive negotiation, α = 0.76.

Couple Satisfaction Index 4 (CSI-4) [34]. The CSI-4 was used to assess the participants’ satisfaction with their romantic relationship. The scale was previously adapted for use in Romanian [35]. Four items are included in the scale. The authors created the CSI scale by verifying the already existing measures of satisfaction and selecting the best items from them. Participants had to indicate how happy they are in their current romantic relationship using a Likert-type scale with seven points for one item (“Please indicate the degree of happiness, all things considered, of your relationship”) and a Likert-type scale with six points for the others (e.g., “I have a warm and comfortable relationship with my partner”). Since the scale uses items with different measurement points, the total score was computed by summing the items. In this study, α = 0.91, indicating a very good internal consistency. 

Conflict scale [36]. The scale measured the level of conflict in the participants’ romantic relationship. The instrument contained six items (e.g., “There is a lot of conflict in my relationship”). For each item, the participants had to choose an answer from 1 (strongly disagree) to 7 (strongly agree). The total score was obtained by averaging the items. The instrument demonstrated a very good internal consistency, α = 0.85.

The scales measuring negotiation tactics and couple conflict that did not have Romanian adaptations were translated using the back-translation method. 

### 2.4. Statistical Analyses

First, I used SPSS 21 to compute the descriptive statistics and the correlational analyses. Second, several mediation analyses were performed to verify whether the negotiation tactics used by individuals in their romantic relationship mediated the relationship between the participants’ sense of relational entitlement and their couple satisfaction and conflict. Model 4 from Process, an SPSS macro, was used for this analysis [37]. Bootstrapping with 5000 re-samples was used to obtain parameter estimates of the specific indirect effects. The 95% confidence intervals (CIs) were used to determine whether these effects were statistically significant: if the 95% CI did not contain zero, then the indirect effect was considered statistically significant and mediation has been demonstrated.

## 3. Results

### 3.1. Preliminary Analyses

I assessed the normality of the distribution for each variable using the Skewness and Kurtosis measures. The values were within the limits proposed by Kim [38] when working with samples larger than 300 participants. I used Pearson product correlations to test the associations between the variables. Age and the length of the relationship were also included in the analysis. Table 1 presents these results as well as the means, standard deviations and values for Skewness and Kurtosis for the variables. Having an excessive sense of relational entitlement is significantly and negatively associated with using more competitive negotiation tactics and with more couple conflict. On the contrary, it is also significantly associated with lower couple satisfaction. A similar pattern of correlations was observed for the restricted sense of relational entitlement, but the strength of the associations was lower. Using cooperative negotiation tactics was significantly associated with higher satisfaction and lower conflict. Using competitive negotiation tactics was significantly associated with lower satisfaction and higher conflict. The length of the relationship was significantly and negatively linked with couple satisfaction. Age was significantly and positively related to the use of competitive negotiation tactics.

A series of independent sample T tests was used to verify the differences in the variables’ levels based on gender and cohabitation status. The results revealed that men, compared with women, reported significantly higher levels of couple satisfaction (t = 3.05; *p* = 0.002) and competitive negotiation tactics (t = 3.5; *p* < 0.001), as well as significantly lower levels of cooperative negotiation tactics (t = −2.70; *p* = 0.007) and restricted sense of relational entitlement (t = −5.28; *p* < 0.001). The individuals who were not cohabitating with their partners, compared with those who were, reported higher levels of couple conflict (t = 2.59; *p* = 0.01), excessive (t = 2.25; *p* = 0.02) and restricted sense of relational entitlement (t = 3.81; *p* < 0.001). 

### 3.2. Mediation Analyses

I intended to compute a series of mediation analyses where either excessive or restricted senses of relational entitlement were introduced as predictors, cooperative and competitive negotiation tactics were used as mediators and either couple satisfaction or conflict served as outcomes. For all models, the participants’ gender, their cohabitation status, their age and the length of their relationships were used as control variables. 

The first model included an excessive sense of relational entitlement as the predictor, the use of competitive negotiation tactics as a mediator and couple satisfaction as the outcome. The effect of the excessive sense of relational entitlement on the use of competitive negotiation tactics was significant (β = 0.41; *p* < 0.001), as well as its effect on couple satisfaction (β = −0.61; *p* < 0.001). The effect of the mediator on the outcome was not significant (β = −0.03; *p* = 0.33). This led to a non-significant indirect effect (β = −0.01; CI (−0.04; 0.01). Thus, the mediation hypothesis was not supported. Additionally, when using couple conflict as an outcome, the effect the mediator had on it was not significant (β = 0.01; *p* = 0.52). The indirect effect was also not significant (β = 0.007; CI (−0.01; 0.03)).

**Figure 1:**
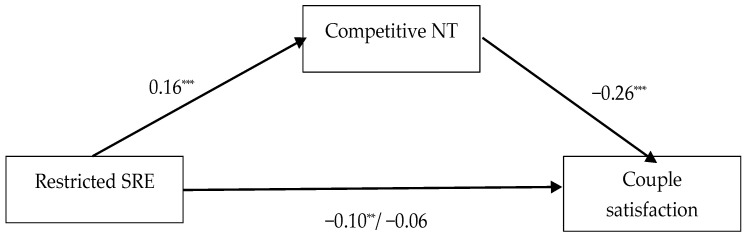
Total and direct effects of restricted sense of relational entitlement (SRE) on couple satisfaction, with competitive negotiation tactics (NT) as mediator. The values represent standardized regression coefficients. On the path between restricted SRE and couple satisfaction, the first value represents the total effect and the second value represents the direct effect. The effects of the controlled variables (age, gender, relationship length and cohabitation status) are not presented for brevity. ** *p* < 0.01; *** *p* < 0.001.

The third model included a restricted sense of relational entitlement as the predictor, the use of competitive negotiation tactics as the mediator and couple satisfaction as the outcome (see Figure 1). Restricted entitlement had a significant effect on the mediator (β = 0.16; *p* < 0.001). Its total effect on the outcome was also significant (β = −0.10; *p* = 0.005). Using competitive negotiation tactics had a significant effect on couple satisfaction (β = −0.26; *p* < 0.001). The indirect effect of the sense of restricted entitlement on couple satisfaction was significant (β = −0.04; CI (−0.06; −0.02)), but its direct effect became non-significant after the introduction of the mediator (β = −0.06; *p* = 0.09). As for the control variables, some significant effects were found. The participants’ gender had negative effects on the use of competitive negotiation tactics (β = −0.11; *p* = 0.004) and couple satisfaction (β = −0.16; *p* < 0.001). Additionally, their relationship duration had a negative effect on couple satisfaction (β = −0.09; *p* = 0.02), while their cohabitation status had a positive one (β = 0.11; *p* = 0.006).

**Figure 2:**
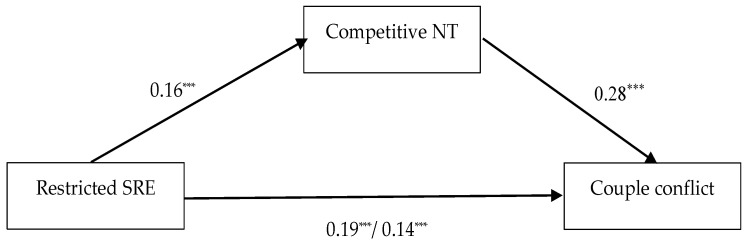
Total and direct effects of restricted sense of relational entitlement (SRE) on couple conflict, with competitive negotiation tactics (NT) as mediator. The values represent standardized regression coefficients. On the path between restricted SRE and couple conflict, the first value represents the total effect and the second value represents the direct effect. The effects of the controlled variables (age, gender, relationship length and cohabitation status) are not presented for brevity. *** *p* < 0.001.

In the fourth model, couple conflict was used as an outcome (see Figure 2). Restricted entitlement had a significant total effect on the outcome (β = 0.19; *p* < 0.001). Using competitive negotiation tactics had a significant effect on couple conflict (β = 0.28; *p* < 0.001). The indirect effect of the sense of restricted entitlement on couple conflict was significant (β = 0.04; CI (0.02; 0.07)), as well as its direct effect after the introduction of the mediator (β = 0.14; *p* < 0.001). The participants’ cohabitation status had a negative effect on couple conflict (β = −0.08; *p* = 0.04).

**Figure 3:**
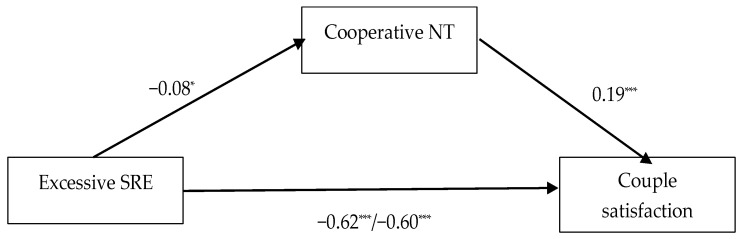
Total and direct effects of excessive sense of relational entitlement (SRE) on couple satisfaction, with cooperative negotiation tactics (NT) as mediator. The values represent standardized regression coefficients. On the path between excessive SRE and couple satisfaction, the first value represents the total effect and the second value represents the direct effect. The effects of the controlled variables (age, gender, relationship length and cohabitation status) are not presented for brevity. * *p* < 0.05; *** *p* < 0.001.

The fifth model included an excessive sense of relational entitlement as the predictor, the use of cooperative negotiation tactics as the mediator and couple satisfaction as the outcome (see Figure 3). Excessive entitlement had a significant effect on the mediator (β = −0.08; *p* = 0.02). Its total effect on the outcome was also significant (β = −0.62; *p* < 0.001). Using cooperative negotiation tactics had a significant effect on couple satisfaction (β = 0.19; *p* < 0.001). The indirect effect of the sense of excessive entitlement on couple satisfaction was significant (β = −0.01; CI (−0.03; −0.002)), and its direct effect remained significant after the introduction of the mediator (β = −0.60; *p* < 0.001). The participants’ gender had a positive effect on their use of cooperative negotiation tactics (β = 0.10; *p* = 0.001).

**Figure 4:**
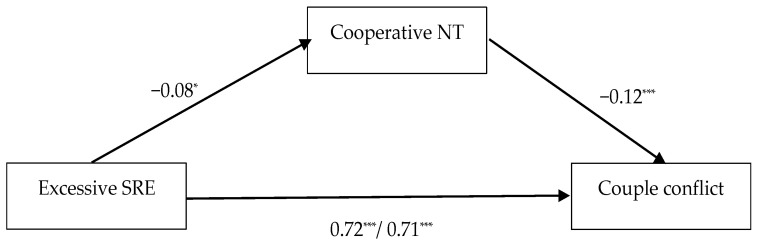
Total and direct effects of excessive sense of relational entitlement (SRE) on couple conflict, with cooperative negotiation tactics (NT) as mediator. The values represent standardized regression coefficients. On the path between excessive SRE and couple conflict, the first value represents the total effect and the second value represents the direct effect. The effects of the controlled variables (age, gender, relationship length and cohabitation status) are not presented for brevity. * *p* < 0.05; *** *p* < 0.001.

In the sixth model, couple conflict was used as an outcome (see Figure 4). Excessive entitlement had significant total effect on the outcome (β = 0.72; *p* < 0.001). Using cooperative negotiation tactics had a significant effect on couple conflict (β = −0.12; *p* < 0.001). The indirect effect of a sense of excessive entitlement on couple conflict was significant (β = 0.01; CI (0.001; 0.02)), as well as its direct effect after the introduction of the mediator (β = 0.71; *p* < 0.001).

In models seven and eight, restricted entitlement was introduced as the predictor, the use of cooperative negotiation tactics as the mediator and either couple satisfaction (model seven) or couple conflict (model eight) as the outcomes. Restricted entitlement did not have a significant effect on the mediator (β = −0.01; *p* = 61). The indirect effects of restricted entitlement on couple satisfaction (β = −0.004; CI (−0.02; 0.01)) and couple conflict (β = 0.003; CI (−0.01; 0.01)) were not significant.

## 4. Discussion

This is one of the first studies to link the sense of relational entitlement, viewed here as an interpersonal difference, the negotiation tactics used in one’s romantic relationship and several couple-related outcomes. I mainly aimed to test the mediating role of couple negotiation tactics (competitive and cooperative) between excessive and restricted SRE, and couple satisfaction and conflict. The results partially supported the assumptions.

For the first two hypotheses, I proposed that higher SRE (excessive or restricted) would be associated with lower levels of couple satisfaction and higher levels of couple conflict. Both hypotheses were confirmed. The relationships were stronger for excessive SRE compared with restricted SRE. All these results confirm previous findings [6,16,18,19], thus adding to the literature that presents high levels of SRE as an important inhibitor of fulfilling couple relationships. Both forms of maladaptive SRE result from unmet childhood needs, which lead to disrupting degrees of need-fulfillment seeking during adulthood [14]. Since romantic relationships are the main life domain where adults express their emotional needs, people who reported deficient close bonds in early childhood tend to also report them in later relationships. Individuals with high levels of excessive SRE exaggerate their needs and react negatively when these are not met by their partners. They are eager to observe and punish even minor transgressions from their partner. Since their partners are rarely able to adequately respond to their highly demanding needs, excessively entitled individuals become less satisfied with their relationships. Moreover, excessive entitlement can inflate self-image goals [39], and when these are not attained, the partner can be blamed, thus creating higher conflict in the relationship. Similarly, individuals with restricted SRE might feel dissatisfied and report higher conflict because they consider that their partners are not attentive to their needs [6,40]. However, their own modest and extremely cautious manner of interacting acts as a cause for such unresponsive behavior.

The mechanism linking maladaptive SRE and couple outcomes is explored more in-depth in this study. The mediation models show that excessive SRE is positively associated with the use of competitive negotiation and negatively with the use of cooperative negotiation. These results support the findings of Williams and collaborators [21], which showed that people who are more vigilant towards relationship transgression (a facet of excessive entitlement) also use more dominance and less compromise in their conflict negotiations. A novel result of this study is that restricted SRE was positively associated only with the use of competitive negotiation, which partially contradicts previous research [21]. The previous study found that individuals with restricted SRE were more likely to use compromise. 

One important finding is that the use of competitive negotiation tactics mediates the relationship between restricted SRE and couple outcomes. Based on their early negative experiences with their caregivers, individuals with restricted SRE tend to see themselves as “worthless, inadequate and defective”, yet they possess “masochistic characteristics” [41] (p. 226). Thus, using more competitive negotiation tactics (a negotiation style where one side must win and the other must lose) only allows the individuals, when losing, to reinforce their beliefs that something is wrong with them. Additionally, restricted SRE is positively linked with an avoidant attachment style and seems to share similar developmental conditions [6,40]. Since avoidant individuals use more non-cooperative, dominating conflict resolution strategies, one might also expect a similar response from those with high levels of restricted entitlement. However, using competitive negotiation was shown to be detrimental to one’s relationship satisfaction [23,25]. Similarly, past research has shown that zero-sum beliefs are associated with lower life satisfaction [42]. Thus, people with high levels of restricted SRE presented lower levels of couple satisfaction and higher levels of couple conflict, and one linking mechanism was their use of competitive negotiation tactics. Additionally, through a decrease in the use of cooperative negotiation, higher levels of excessive SRE were associated with lower couple satisfaction and higher couple conflict. Individuals with high entitlement scores can prefer short-term personal goals over long-term development of the couple [43]. They can, as well, present an individualistic orientation, thus being more prone to use threats, bargaining and other types of unethical negotiation [10,43]. Consequently, overly concentrating on winning in any argument and choosing self-benefit instead of collective good can lead individuals to lower levels of relational happiness. 

These findings have important implications for couples therapy as well as for the mental health of the individuals involved in couple relationships. Firstly, as shown by previous studies, negotiation is an important aspect of couple functioning and is relevant in various domains such as parenting, work and even technology use [23,44,45]. Moreover, negotiation can be trained in couples therapy [8]. This study shows that the negotiation tactics used by individuals can be an outcome of some internal aspects such as the sense of relational entitlement. Thus, integrating this knowledge in the field of couple and family therapy is essential to develop more comprehensive programs that account for differences in relational entitlement, especially when educating couples regarding the best ways to use negotiation. Secondly, knowing why some people are more prone to using maladaptive negotiation tactics can have larger implications beyond the well-being of the couple. Both individual case studies and empirical studies have shown that couples therapy is efficient when dealing with issues such as depression [46,47,48]. Moreover, since couple satisfaction acts as a predictor for depression, family therapy can be used to ensure higher levels of couple, as well as individual, functioning. Thus, educating positive couple interactions (such as using fewer competitive negotiation tactics) while also taking into account the individual issues affecting them can lead to better-suited therapeutic processes and more efficient ways to protect mental health. 

On another interesting note, this study shows that SRE is an antecedent of couple conflict. Previous studies evidenced that interparental conflict is a predictor of the development of maladaptive SRE [49]. Although indirectly, these two studies present some evidence regarding the transmission of couple conflict from one generation to the other. 

Despite advancing the literature and having important practical implications, this study is not without limitations. Firstly, it proposes a cross-sectional investigation using self-reported questionnaires. To truly test the mediation hypothesis, longitudinal studies are needed. Additionally, more recent studies assess the partners’ behaviors by using recordings of the couples’ conflicts [50,51]. With a similar methodology, future studies can investigate the negotiation tactics used by the partners during a conflictual interaction and their effects on conflict resolution. Finally, the use of negotiation seems to differ based on age and cultural characteristics. Past studies showed that younger individuals and those coming from cultures higher in individualism and masculinity tend to use more unethical or competitive tactics [52]. Since in this sample, the participants were rather homogenous in age and came from only one culture, these results need to be confirmed by research with more diverse participants.

## 5. Conclusions

The main aim of this study was to test the mediation role of negotiation tactics in the relationship between the sense of relational entitlement and couple satisfaction and conflict. The results showed that restricted SRE is related to higher levels of competitive negotiation use, while excessive SRE is linked to lower levels of cooperative negotiation use. Both negotiation tactics mediated the link between SRE and couple satisfaction, as well as SRE and couple conflict. The research shows that SRE is a significant antecedent of negotiation behavior and how this couple process impacts various relational outcomes.

## Figures and Tables

**Table 1:** Means, standard deviations and correlations between the study’s variables.

	M	SD	SK	Kr	1	2	3	4	5	6	7
1. Excessive SRE	1.87	0.82	1.25	1.32	0.88						
2. Restricted SRE	2.51	1.08	0.31	−0.83	0.31 ***	0.79					
3. Cooperative NT	4.09	0.55	−0.70	0.90	−0.06	−0.01	0.65				
4. Competitive NT	1.96	0.71	0.72	0.12	0.37 ***	0.16 ***	−0.22 ***	0.76			
5. Couple satisfaction	21.95	3.15	−1.31	1.52	−0.63 ***	−0.10 **	0.23 ***	−0.26 ***	0.91		
6. Couple conflict	2.32	1.13	1.13	1.03	0.70 ***	0.19 ***	−0.18 ***	0.30 ***	−0.69 ***	0.85	
7. Age	22.97	3.54			−0.03	−0.06	−0.05	0.08 *	−0.02	−0.03	-
8. Relationship length	36.31	32.49			0.03	−0.10 **	−0.05	0.04	−0.08 *	0.01	0.43 ***

Note: SK = Skewness; Kr = Kurtosis; SRE = sense of relational entitlement; NT = negotiation tactics; *** *p* < 0.001; ** *p* < 0.01; * *p* < 0.05. Cronbach’s alpha for each scale is shown on the diagonal.

## Data Availability

The data presented in this study are available on request from the corresponding author.
